# The MRGPRX2 paradigm shift: Redefining mast cell activation pathways in chronic urticaria 

**DOI:** 10.5414/ALX02618E

**Published:** 2026-04-23

**Authors:** Kun Wu, Junlin Liu

**Affiliations:** Department of Dermatology and Venereology, The Second Affiliated Hospital of Hainan Medical University, Haikou, Hainan, China

**Keywords:** chronic urticaria, Mas-related G protein-coupled receptor X2, mast cells

## Abstract

Chronic urticaria (CU) is a skin disease characterized by recurrent episodes of vascular dilation and increased permeability of the cutaneous and mucosal microvasculature. Although antihistamines and omalizumab remain first-line and second-line therapies, respectively, a significant proportion of patients develop recalcitrant disease phenotypes, highlighting critical unmet needs for innovative therapeutic paradigms. In recent years, emerging insights into Mas-related G protein-coupled receptor X2 (MRGPRX2) have revealed transformative perspectives for elucidating the pathobiology of refractory CU. As a class A G protein-coupled receptor (GPCR) that is predominantly localized to mast cells, MRGPRX2 orchestrates non-IgE-mediated mast cell degranulation through its pluripotent ligand recognition capacity, engaging diverse exogenous cationic compounds, neuropeptides, and certain pharmacological agents. This comprehensive review evaluates recent advancements in deciphering the mechanistic contributions of MRGPRX2 to CU pathogenesis, with the ultimate aim of informing the development of precision diagnostic and therapeutic frameworks for CU management.

## Introduction 

### Aberrant mast cell activation: The central pathogenic mechanism 

Chronic urticaria (CU) is formally characterized as a persistent, immune-mediated dermatological disorder manifesting as recurrent wheals, pruritus, and angioedema persisting for ≥ 6 weeks [1]. The core pathophysiological mechanism of CU stems from the dysregulated activation and pathological degranulation of cutaneous mast cells (MCs). This aberrant cellular behavior triggers the release of preformed and newly synthesized mediators, including histamine, tryptase, leukotrienes, prostaglandins, and a spectrum of cytokines and chemokines. These mediators collectively orchestrate a cascade of pathobiological events, including dermal microvascular hyperpermeability, nociceptor sensitization, inflammatory cell infiltration (e.g., eosinophils, neutrophils, lymphocytes), and endothelial activation, culminating in characteristic clinical manifestations [2, 3]. 

### Limitations of conventional therapies: Heterogeneity in treatment responsiveness 

**Figure 1.**
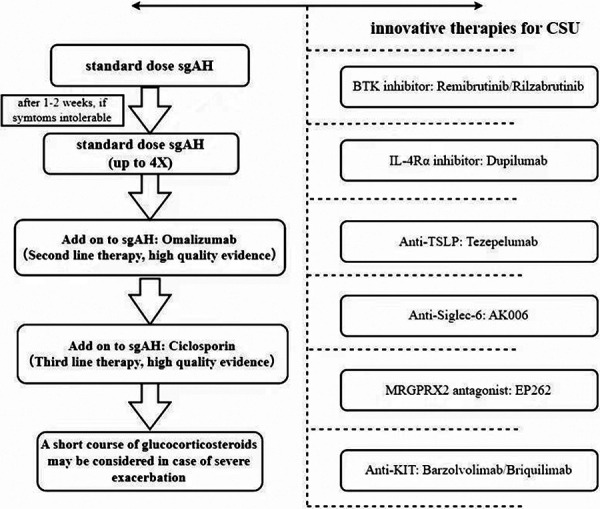
Illustrative overview of international authoritative guidelines and innovative therapies for hronic spontaneous urticaria: This overview is based on evidence-based consensus from EAACI/GA^2^LEN/EuroGuiDerm/APAAACI and clinical trial evidence [5, 6] (This figure was drawn by Figdraw). TSLP = thymic stromal lymphopoietin; Siglec-6 = sialic acid-binding immunoglobulin-like lectin-6.

Contemporary epidemiological evidence indicates that 40 – 50% of CU patients experience a suboptimal response to antihistamine monotherapy at standard doses, culminating in persistent symptoms and clinically significant deterioration in health-related quality of life (HRQoL) [4]. Intriguingly, although a subset of 35 – 40% of CU patients exhibit elevated serum immunoglobulin E (IgE) levels, a paradoxical subgroup within this population demonstrates inadequate clinical responsiveness to IgE-targeted biological therapies, such as omalizumab. This disparity in therapeutic efficacy strongly implicates the significant contribution of non-IgE-mediated mechanisms to MC activation pathways in CU pathogenesis, particularly in refractory cases. The current therapeutic paradigm, as delineated by the international EAACI/GA^2^LEN/EDF/WAO guidelines, mandates a stratified pharmacotherapeutic algorithm for refractory cases (Figure 1): first-line therapy involves a second-generation H1-antihistamine. For suboptimal responders, stepwise dose escalation of first-line agents to 4-fold the standard dosage is recommended. Omalizumab is designated second-line therapy, with cyclosporine reserved as a third-line intervention for patients refractory to preceding treatments [5]. However, emerging clinical data reveal persistent therapeutic inadequacies, with 20 – 30% of CU patients maintaining recalcitrant disease progression despite optimized treatment protocols adhering to these guidelines. Furthermore, omalizumab has durable response rates in only 60 – 70% of recipients during extended therapeutic regimens [6, 7]. This persistent therapeutic gap underscores the imperative to delineate alternative MC activation mechanisms, particularly those transduced via non-IgE receptor signaling cascades such as the Mas-related G protein-coupled receptor X2 (MRGPRX2) pathway. 

### Molecular determinants of MRGPRX2 and its role in MC activation heterogeneity 

The human MRGPR family, comprising eight distinct subtypes (MRGPRX1-X4 and MRGPRD-G), constitutes a phylogenetically conserved class of sensory receptors that mediate multifaceted host defense mechanisms through neuroimmunological integration. These receptors play critical pathophysiological roles in neuroimmune crosstalk, including pruriceptive signal transduction, nociceptive sensitization cascades, the modulation of inflammatory responses, and the maintenance of innate immune homeostasis [8]. Among these subtypes, MRGPRX2 has gained prominence in cutaneous immunity owing to its predominant expression in MCs and diverse signaling capabilities. 

Mechanistically, in contrast to the canonical high-affinity IgE receptor (FcεRI)-mediated cascade, MRGPRX2 triggers rapid G protein-coupled MC activation. This pathway involves a self-limiting signaling paradigm characterized by transience and self-limitation [9, 10]. The unique structural permissiveness of the receptor enables the promiscuous engagement of heterogeneous agonist classes, including pharmacological ligands (e.g., neuromuscular blocking drugs, cationic ligands), xenobiotic compounds (synthetic food additives/preservatives), and endogenous neuropeptides. This ligand promiscuity underpins MRGPRX2’s ability to elicit pseudoallergic inflammatory responses through the coordinated release of preformed cytokines, de novo synthesized chemokines, and vasoactive mediators. The resulting pathophysiological environment elicits clinical manifestations that phenocopy classical type I IgE-mediated hypersensitivity reactions, establishing MRGPRX2 as a molecular lynchpin in drug-induced pseudoallergic reactions [11], neurogenic inflammation paradigms, and potentially, aspects of chronic inflammatory skin conditions. 

### Neuroimmune crosstalk in pathological cascades: Bridging receptor activation to the pruritic–inflammatory cycle 

Emerging insights into CU pathophysiology reveal that dysregulation of the MRGPRX2 axis impairs MC homeostatic regulation while concurrently activating pruriceptive neuronal populations (e.g., TRPV1 neurons) via bidirectional neuroimmune crosstalk [12]. This interaction perpetuates a self-sustaining pruritic‒inflammatory cycle central to chronic disease pathogenesis. Convergent evidence from multidisciplinary investigations has revealed a distinct degranulation profile associated with MRGPRX2 activation compared with FcεRI triggering. MRGPRX2 engagement is characterized predominantly by tryptase release, in contrast with the relatively attenuated secretion of histamine and serotonin [13]. Substantiating these mechanistic observations, rigorous biomarker analyses revealed significant increases in both MRGPRX2 receptor density (mRNA and protein) and the bioavailability of endogenous ligands, particularly neuropeptide substance P (SP), major basic protein (MBP), and eosinophil peroxidase (EPO), within the lesional skin microenvironments and systemic circulation of CU patients [14, 15]. 

These pathophysiological correlations not only elucidate the critical role of neuroimmune microenvironmental perturbation in CU pathogenesis but also establish quantitative parameters of MRGPRX2 signaling dynamics (e.g., receptor expression levels and ligand concentrations) and ligand‒receptor stoichiometry as novel molecular biomarkers. Such metrics hold significant potential for clinical application in stratifying disease activity, predicting treatment response, monitoring treatment efficacy, and ultimately enabling personalized treatment strategies. 

## Potential activators of MRGPRX2 (Table 1) 

### Neurogenic activators: Mechanistic insights into substance P and hemokinin-1 

As a prototypic proinflammatory neuropeptide of the tachykinin family, SP critically engages psychocutaneous stress pathways [16] and functions as a high-affinity endogenous agonist for MRGPRX2. Its release from sensory nerve terminals is triggered by diverse stimuli, including neurogenic stress, physical insults, and inflammatory cascades. Mechanistically, SP orchestrates MC degranulation while concomitantly potentiating the chemotactic recruitment of macrophages. Collectively, this neuroimmunomodulatory axis drives a self-sustaining pathophysiological triad encompassing cutaneous barrier compromise, afferent neuronal hypersensitization, and dysregulated innate immunity. 

Clinical biomarker profiling revealed significantly elevated circulating SP levels in CU patients compared with healthy controls. Moreover, the SP concentration strongly positively correlated with the Urticaria Activity Score over 7 days (UAS7) [17]. Intriguingly, no significant differences in other neuropeptides, such as calcitonin gene-related peptide (CGRP), vasoactive intestinal peptide (VIP), neuropeptide Y (NPY), or nerve growth factor (NGF), were detected between the two groups. Flow cytometry revealed increased surface density of tachykinin receptor 1 (NK-1) on Th2-polarized basophils in the CU, suggesting novel immunoregulatory crosstalk between neurogenic inflammation and adaptive immune responses. Mechanistic studies further delineate SP-mediated transcriptional upregulation of corticotropin-releasing hormone receptor 1 (CRH-1) in MCs, thereby creating a bidirectional interface between hypothalamic pituitary adrenal (HPA) axis activation and sympathetic-adrenomedullary signaling. This molecularly couples psychosocial stress with cutaneous neuroinflammation, potentially explaining stress-induced exacerbations in individuals with CU [8]. 

Hemokinin-1 (HK-1), a more recently identified tachykinin family member that shares approximately 64% sequence homology with SP, exhibits intriguing functional divergence within MRGPRX2 signaling pathways. Nishimori et al. [18] demonstrated through calcium flux assays that HK-1, an agonist, can induce MRGPRX2 desensitization, resulting in > 60% inhibition of subsequent SP-mediated MC degranulation. This regulatory mechanism potentially involves β-arrestin-initiated receptor internalization and uncoupling from G proteins, indicating the dual modulatory role of HK-1 in neurogenic inflammation [18]. The relative abundance and spatial‒temporal release patterns of SP and HK-1 in the skin microenvironment may therefore critically influence the net outcome of MRGPRX2 signaling. 

### Eosinophil-derived activating proteins: The MBP/EPO/ECP signaling nexus 

The pathological persistence of MRGPRX2 activation in the CU is partially mediated by aberrant deposition of eosinophilic granular protein within the cutaneous microenvironment. In patients with CU, autoantibodies targeting the low-affinity IgE receptor (FcɛRII/CD23) expressed on eosinophils increase degranulation via activation of the PI3K/Akt signaling pathway, leading to increased release of the cationic granule proteins MBP and EPO [19]. Immunohistochemical quantification revealed significantly elevated densities of MBP^+^ and EPO^+^ cells within lesional versus nonlesional skin, with protein expression intensity showing statistically significant positive correlations with validated pruritus severity indices. Eosinophil cationic protein (ECP) has dual clinical relevance. First, serum ECP levels serve as a dynamic biomarker reflecting overall eosinophil activation and disease activity in CUs. Second, and more critically, ECP functions as a direct MRGPRX2 agonist through stereospecific binding to its extracellular domain. This ligand‒receptor interaction initiates MC-mediated secretory cascades, including the release of alarmins such as interleukin-33 (IL-33) and thymic stromal lymphopoietin (TSLP), through IgE/FcεRI-independent pathways [20, 21]. 

### Pharmacological activators: polypharmacological profiling 

The pathophysiology of opioid-induced chronic pruritus, a well-established adverse effect, involves dual dysregulation: direct effects on neural itch pathways via μ-opioid receptor (MOR)/κ-opioid receptor (KOR) imbalance combined with MRGPRX2-dependent MCs. Pharmacodynamic analyses revealed that certain cationic synthetic opioids (e.g., morphine and codeine) exhibit up to 10-fold greater potency at MRGPRX2 receptors than at classical μ-opioid receptors in recombinant assays. This activation induces rapid intracellular calcium flux in laboratory allergic disease 2 human mast cells (LAD2), triggering substantial histamine release [22]. These findings suggest that opioid metabolites or parent opioids may induce receptor priming or sensitization via epigenetic modifications of MRGPRX2 promoter regions or alterations in receptor trafficking. 

Notably, drug hypersensitivity reactions in CU patients manifest as significantly reduced cutaneous reactivity thresholds to specific agents. For example, heightened responses to the bradykinin B2 receptor antagonist icatibant and the benzylisoquinoline neuromuscular blocking agent atracurium compared with those of controls have been reported. This hypersensitivity is mechanistically driven by pathological MRGPRX2 overexpression and ligand-binding pocket sensitization within the lesional skin microenvironment [23]. Furthermore, comprehensive structural and functional analyses have identified several additional drug classes as direct MRGPRX2 agonists, including fluoroquinolones (e.g., ciprofloxacin), glycopeptides (e.g., vancomycin), and tyrosine kinase inhibitors (e.g., imatinib mesylate) [24]. 

The plant-derived alkaloid sinomenine stimulates the release of monocyte chemoattractant protein-1 (MCP-1), IL-8, and macrophage inflammatory protein-1α (MIP-1α). The proedema effects of MRGPRX2 in experimental models are attenuated by MRGPRX2 antagonists. Flavonoids such as baicalin exhibit concentration-dependent biphasic modulation: MC activation at low concentrations (< 10 μM) contrasts with degranulation inhibition at elevated concentrations (> 50 μM). This paradoxical behavior likely arises from dynamic allosteric regulation of receptor conformations, suggesting context-dependent partial agonism or allosteric modulation [3, 25]. Specifically, the MRGPRX2/Mrgprb2-dependent pseudo-allergic effect of baicalin has been characterized in vitro using rat basophilic leukemia (RBL-2H3) cells and Mrgprb2-transfected HEK293 cells, and confirmed in vivo in murine models [26]. 

### Novel activators: Mechanistic discoveries and therapeutic implications 

Accumulating experimental evidence has demonstrated the critical pathophysiological involvement of platelet-activating factor (PAF) in CU pathogenesis. PAF is a proinflammatory phospholipid synthesized and secreted by MCs, monocytes, and tissue macrophages, among others. Comparative analyses revealed significantly elevated serum PAF concentrations in CSU (chronic spontaneous urticaria) patients compared with healthy controls (p < 0.001), demonstrating strong positive correlations with disease severity as quantified by UAS7 [27]. Proteomic profiling has revealed tissue-specific differential expression patterns of PAFR mRNA between human cutaneous and pulmonary MCs, highlighting its preferential engagement in dermal inflammatory pathways. Notably, persistent PAF elevation often persists in antihistamine-refractory CU patients, establishing its potential as both a biomarker for therapeutic resistance and a direct molecular mediator of recalcitrant disease states via the activation of MCs (potentially through MRGPRX2 or other receptors) and other immune cells [28]. 

Recent translational research has identified anti-heat shock protein 10 (HSP10) IgG autoantibodies as potential novel activators of MRGPRX2, with structural analyses suggesting a potential mechanism involving molecular mimicry. HSPs, which function as molecular chaperones in protein folding and cellular stress responses, play critical immunoregulatory roles. Clinical investigations in severe CSU cohorts revealed an inverse correlation between circulating anti-HSP10 IgG titers and endogenous HSP10 concentrations, concomitant with persistent PAF elevation. This pathogenic triad suggests that anti-HSP10 antibodies may either disrupt HSP10 homeostatic functions or directly cross-link MRGPRX2 via molecular mimicry, thereby initiating MC degranulation. Epigenetic mechanistic studies further delineate the mechanism by which HSP10 governs MC activation thresholds: HSP10 epigenetically suppresses MRGPRX2 expression through miR-101-5p-mediated post-transcriptional silencing, which is achieved via direct targeting of the HSP10 3’-untranslated region (3’-UTR) to modulate transcript stability and translational efficiency [29]. Collectively, these findings suggest two synergistic therapeutic modalities: first, immunomodulatory targeting of the HSP10–MRGPRX2 axis, and second, miRNA-based precision engineering to restore MC stability, representing transformative paradigms in urticaria management. 

## MRGPRX2 triggered non-IgE-mediated MC activation pathways (Figure 2) 

### Canonical G protein-coupled signaling cascades 

The molecular basis of MRGPRX2-mediated MC degranulation involves tightly coordinated interplay among G protein-coupled signaling cascades, calcium dynamics, and kinase phosphorylation. Structural characterization of MRGPRX2 reveals a unique ligand-binding interface featuring a shallow, solvent-exposed pocket within the receptor’s N-terminal domain (NTD), extracellular loops (ECLs), and transmembrane helices (TMs). This structural plasticity underlies the promiscuous ligand-binding capacity, enabling the recognition of diverse endogenous and exogenous agonists [30]. Ligand engagement at the NTD initiates canonical Gα_q_ protein coupling, triggering phospholipase C-β (PLC-β) activation and subsequent hydrolysis of phosphatidylinositol 4,5-bisphosphate (PIP2) into inositol 1,4,5-trisphosphate (IP_3_) and diacylglycerol (DAG). IP_3_ binding to its receptors on the endoplasmic reticulum (ER) mediates calcium store depletion, inducing rapid cytosolic Ca^2+^ mobilization, which directly drives secretory granule exocytosis [31]. 

Concurrently, DAG activates protein kinase C (PKC) isoforms. PKC activation contributes to multiple downstream effects, including the modulation of ion channels, cytoskeletal reorganization facilitating degranulation, and the activation of transcription factors. Pertussis toxin (PTX)-sensitive Gα_i_ coupling inhibits adenylyl cyclase, reducing intracellular cyclic AMP (cAMP) levels, which may disinhibit certain activation pathways. Critically, Gα_i_ signaling amplifies downstream effector pathways via the activation of Src family kinases, leading to the phosphorylation and activation of extracellular signal-regulated kinases 1/2 (ERK1/2), phosphoinositide 3-kinase (PI3K)/Akt, and p38 mitogen-activated protein kinase (MAPK) [3]. This integrated signaling network coordinates not only the immediate release of preformed mediators but also the transcriptional and posttranslational regulation of de novo synthesized proinflammatory mediators, such as IL-31, prostaglandin E_2_ (PGE_2_), and leukotriene C_4_ (LTC_4_). Consequently, it links immediate degranulation events with sustained inflammatory responses and tissue remodeling. 

### Orai/CRAC channel-dependent inflammatory modulation 

Accumulating experimental evidence supports a critical regulatory role for Orai/CRAC channels in MRGPRX2-mediated MC degranulation and proinflammatory cytokine biosynthesis. Chaki et al. [32] demonstrated that Orai-dependent store-operated calcium entry (SOCE) is essential for antigen-evoked Ca^2+^ mobilization in MCs and directly modulates TNF-α, IL-8, and MIP-1α production downstream of MRGPRX2 signaling. These findings implicate Orai/CRAC channels in MC-mediated inflammatory pathologies and suggest their potential as therapeutic targets [32]. Further mechanistic studies confirmed that pharmacological inhibition via Synta-66, a selective CRAC channel blocker, results in multimodal suppressive effects. Synta-66 achieves concomitant inhibition of the ERK1/2 and Akt phosphorylation cascades, thereby disrupting both calcium-dependent degranulation and cytokine transcriptional programs [33]. This dual-pathway intervention strategy highlights previously unrecognized dimensions of CRAC channel pharmacology while providing a mechanistic rationale for targeted therapeutic development in MC-mediated inflammatory diseases. 

### Lysyl-tRNA synthetase-microphthalmia-associated transcription factor transcriptional regulatory axis 

In addition to facilitating canonical plasma membrane-initiated signaling and calcium cascades, MRGPRX2 activation engages sophisticated nuclear signaling pathways to regulate gene expression programs essential for sustaining inflammation and MC function. The lysyl-tRNA synthetase (LysRS)-microphthalmia-associated transcription factor (MITF) pathway has been identified as a critical molecular determinant orchestrating MRGPRX2-mediated MC activation and mediator production. Mechanistically, this signaling pathway is regulated by MRGPRX2-induced transient ERK1/2 phosphorylation. Following agonist binding (e.g., SP), activated ERK1/2 phosphorylates LysRS, a component of the multi-tRNA synthetase complex (MSC), leading to its dissociation from the MSC and subsequent nuclear translocation. Phosphorylation triggers a conformational change in LysRS, leading to its dissociation from the MSC and subsequent nuclear translocation [34]. 

Furthermore, MRGPRX2 signaling orchestrates the posttranslational modification of MITF, increasing its phosphorylation. This modification potentiates the transcriptional competence and chromatin remodeling capacity of MITF. Notably, genetic ablation or knockdown of MITF significantly impaired SP-MRGPRX2-induced intracellular Ca^2+^ mobilization and degranulation, underscoring its indispensable role in this pathway. MITF is recognized as the master transcriptional regulator of MC biology. Its expression and activity are reciprocally regulated through a bidirectional network with the KIT receptor tyrosine kinase signaling pathway, which is essential for MC development and survival. Specifically, KIT activation inhibits MITF expression via microRNA pathways (e.g., miR-539/miR-381), whereas MITF conversely maintains KIT expression through positive feedback mechanisms [35]. This establishes a dynamic homeostatic regulatory circuit essential for MC differentiation and function, which may also contribute to MC hyperreactivity in disease contexts such as CU. Consequently, dysregulation of the MRGPRX2-LysRS-MITF axis fundamentally alters MC responsiveness. 

## MRGPRX2-specific inhibitors: emerging therapeutic paradigms 

The recognition of the central role of MRGPRX2 in non-IgE-mediated MC activation, particularly in refractory CU, has led to the development of multifaceted interventional strategies targeting this receptor or its downstream signaling pathways. 

Innovative photodynamic precision therapy approaches utilize photosensitizer-conjugated anti-MRGPRX2 antibodies. Following localized near-infrared irradiation, this strategy enables spatially selective ablation of MRGPRX2-overexpressing MCs within cutaneous tissues, representing a significant advance in localized therapeutic precision with minimal off-target effects. At the molecular level, several classes of compounds, including immunomodulatory single-stranded oligonucleotides, DNA analogs, and tripeptide derivatives, have been identified as potent inhibitors of MRGPRX2-driven MC degranulation [36]. 

The frontier of phytochemical research has led to transformative discoveries, with flavonoid derivatives (e.g., fisetin, quercetin, and luteolin), phenolic compounds, and triterpenoid saponins demonstrating potent inhibitory activity [37]. Pioneering studies have elucidated the mechanism of fisetin, revealing its stereospecific binding to MRGPRX2, where it likely functions as a negative allosteric modulator or competitive antagonist. This interaction downregulates critical phosphorylation cascades encompassing Akt, p38 MAPK, NF-κB, and PLC-γ, effectively suppressing Ca^2+^ signaling and inflammatory mediator release while ameliorating urticarial manifestations in murine models [38]. Artemisinic acid and its precursors exhibit dual modulatory effects, as demonstrated by in vitro kinase assays confirming their ability to attenuate MC degranulation and histamine release via the Lyn‒PLC‒p38‒NF‒κB axis. These findings identify Lyn kinase as a pivotal regulatory node in MRGPRX2-triggered pseudoallergic responses [39]. Consequently, Lyn kinase inhibition has emerged as another promising therapeutic strategy. 

Clarithromycin, a semisynthetic macrolide antibiotic, manifests unique immunomodulatory effects beyond its antimicrobial activity. These effects may be partially mediated through interactions involving the CD300f inhibitory receptor immunoreceptor tyrosine-based inhibitory motif domain, potentially modulating MRGPRX2 signaling or MC activation thresholds. Preclinical validation confirmed the concurrent suppression of IgE-mediated hypersensitivity and MRGPRX2-mediated pseudoallergic reactions, with marked alleviation of pruritus in CU models [4]. 

EP262 represents one of the most promising advanced small-molecule antagonists in preclinical and early clinical development. This highly selective MRGPRX2 antagonist exhibits sustained pharmacodynamic efficacy compatible with once-daily oral dosing and effectively inhibits the release of both early-phase (e.g., histamine) and late-phase (e.g., IL-1, IL-4, IL-5, and IL-13) mediators through MRGPRX2-triggered pathways in human MCs. Current clinical development encompasses a phase 2 trial (NCT06077773) assessing EP262 in H1-antihistamine-refractory CSU and a phase 1b study (NCT06050928) assessing its efficacy in patients with chronic inducible urticaria (CIndU). However, a critical development occurred in 2024: the phase 2 trial in CSU was discontinued. The decision was based on safety concerns arising from preclinical toxicity studies, which identified adverse findings unrelated to MRGPRX2 pharmacology at exposures anticipated in humans [40]. This setback underscores the challenges in drug development targeting MRGPRX2 and highlights the necessity for thorough preclinical safety profiling. 

The clinical landscape for MRGPRX2 antagonists continues to evolve. EVO756 represents another novel, orally administered small-molecule antagonist. Translating its earlier preclinical promise, initial clinical data for EVO756 were reported at the European Academy of Dermatology and Venereology (EADV) 2025 Congress. A phase 2a proof-of-concept study evaluated EVO756 in 30 adult patients with CIndU. Participants were administered EVO756 orally for 4 weeks under one of two dosing regimens: 300 mg once daily or 50 mg twice daily. The preliminary results indicated a reduction in pruritus, and the treatment was reported to be well tolerated within this short-term trial period. These early findings, while promising and representing a tangible step forward from the preclinical stage, require confirmation in larger, longer-term, and peer-reviewed clinical trials to fully establish the efficacy and safety profile of EVO756 for MC-mediated disorders [41]. 

## Conclusion 

To provide a clear overview of the experimental evidence underlying the key findings discussed in this review regarding MRGPRX2’s role in CU, the supporting data are categorized by research model in Table 2. In this review, we present the current understanding of the MRGPRX2 signaling axis and its pivotal role in MC-driven pathophysiology, with a particular focus on the CU. A significant limitation in the field remains the heavy reliance on rodent models (e.g., passive cutaneous anaphylaxis) and human MC lines (e.g., LAD2) for mechanistic studies and drug screening. While invaluable, these models incompletely recapitulate the complexity of human CU pathophysiology, particularly concerning chronicity, immune cell interplay, and neuroimmune crosstalk. Furthermore, the intricate interplay between MRGPRX2 signaling and the broader pathogenic network involved in CU-related autoimmune dysregulation (e.g., autoantibodies targeting FcεRI, FcεRII, and thyroid peroxidase), coagulation cascade activation (e.g., thrombin and factor XIIa), complement involvement, and complex inflammatory cell infiltration/crosstalk (eosinophils, basophils, neutrophils, and lymphocytes) requires further investigation. 

These insights highlight several promising therapeutic avenues: the development of agents that combine potent MRGPRX2 antagonism with complementary immunomodulatory or anti-inflammatory functions (e.g., targeting key cytokines such as IL-4, IL-13, and TSLP or intracellular kinases such as Lyn) may offer superior therapeutic efficacy by concurrently modulating multiple nodes within the CU pathogenic network. Elucidating the high-resolution structure of the MRGPRX2 ligand-binding domain via techniques such as cryo-electron microscopy and X-ray crystallography in combination with agonists or antagonists is imperative for the development of highly selective antagonists with optimized binding affinities, pharmacokinetic profiles, and minimal off-target effects. Creating personalized therapeutic prediction models incorporating MRGPRX2 expression levels, genetic polymorphisms, endogenous/exogenous ligand profiles, and potentially autoantibody status could identify patients most likely to benefit from MRGPRX2-targeted therapy versus alternatives (e.g., omalizumab, cyclosporine, or novel biologics). Crucially, understanding the mechanisms by which MRGPRX2 activation contributes to chronic inflammation in CU remains crucial. Future research must focus on elucidating its long-term consequences for the MC phenotype, neuronal sensitization, and tissue remodeling, particularly its role in perpetuating the MC-nerve unit. Exploring the potential roles of MRGPRX2 in systemic manifestations associated with severe CU or comorbidities could further broaden its therapeutic implications. Further investigations into the MRGPRX2 signaling axis, its integration within the complex CU pathogenic network, and the development of targeted therapeutic strategies are warranted to translate mechanistic insights into improved clinical outcomes. 

## Authors’ contributions 

Kun Wu: Writing – original draft. Junlin Liu: Supervision, writing – reviewing and editing. 

## Funding 

This study was financially supported by National Natural Science Foundation of China (81860551). The funder had no influence on the study design, data collection and analysis, the decision to publish, or the preparation of the manuscript. 

## Conflict of interest 

Both authors declare that there is no conflict of interest in relation to this publication. 

**Table 1.** Characteristics of potential agonists of MRGPRX2.

**Classification**	**Activator**	**Human MCs lineage source**	**References**
Neuropeptide	SP	Skin, synovial, cord, LAD2	[15, 23, 31]
	HK-1	LAD2	[18]
Eosinophil granule protein	MBP	Skin, lung, heart, cord blood-derived	[18, 20, 25]
	EPO	Skin	[18, 20, 25]
	ECP	Heart, cord blood-derived	[18, 20, 25]
Phospholipid	PAF	Lung, peripheral blood-derived	[25, 27]
Protein fragment drugs	HSP10	CD34^+^-derived	[25, 29]
	Morphine	Skin, LAD2	[22, 25]
	Codeine	LAD2, CD34^+^-derived	[25]
	Kinin receptor agonists	LAD2	[23, 25]
	NMBAs	LAD2	[23, 25]
	Quinolone antibiotics	LAD2	[23, 25]
	Vancomycin	LAD2	[23, 25]
	Natural remedies	LAD2	[3, 25]

**Figure 2.**
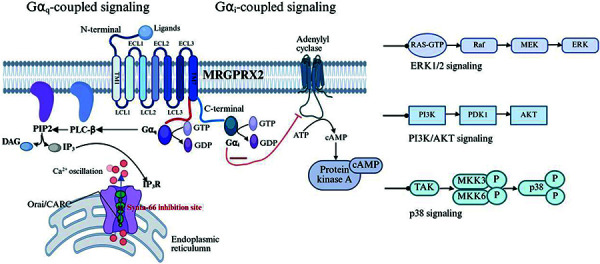
G protein-coupled signaling cascades and Orai/CRAC channel-regulated inflammation after MRGPRX2 activation. (This figure was drawn by Biorender.)

**Table 2.** Categorization of key findings on MRGPRX2 by research model.

**Source of evidence**	**Key findings related to MRGPRX2**	**References**
A. Cell culture models		
Human MC lines (e.g., LAD2)	Predominant tryptase release (vs. FcεRI)	[13]
	Activation and desensitization by neuropeptides (e.g., SP, HK-1)	[15, 18]
	Potent agonism by cationic drugs (e.g., opioids)	[22, 23, 24]
	Key roles of Orai/CRAC channels and LysRS-MITF axis	[32, 34]
Other cell systems	Biphasic modulation by baicalin	[26]
	Epigenetic suppression by HSP10/miR-101-5p	[29]
	Inhibition by artemisinic acid via Lyn kinase	[38]
	In vitro efficacy of inhibitors (e.g., aptamers, clarithromycin)	[4, 36]
B. Animal models		
Rodent models	Validation of MRGPRX2-dependent pseudo-allergy	[3, 25]
	In vivo efficacy of artemisinic acid	[38]
	In vivo protection by inhibitors (e.g., aptamers, clarithromycin)	[4, 36]
	Study of KIT-MITF regulatory network	[34]
C. Human studies		
Clinical biomarker & Tissue analysis	Elevated MRGPRX2 and SP levels correlate with disease activity	[14, 15, 17]
	Enhanced eosinophil degranulation elevates lesional MBP^+^/EPO^+^ cells	[19]
	ECP as agonist and elevated lesional IL-33/TSLP	[20, 21]
	Elevated serum PAF in CSU patients	[27, 28]
	Anti-HSP10 autoantibodies in severe CSU	[29]
Clinical trials	Discontinuation of EP262 phase 2 (toxicity)	[40]
	Preliminary efficacy of EVO756 Phase 2a in CIndU	[41]

## References

[1] Lang DM Chronic Urticaria. N Engl J Med. 2022; 387: 824–831. 36053507 10.1056/NEJMra2120166

[2] Kolkhir P Giménez-Arnau AM Kulthanan K Peter J Metz M Maurer M Urticaria. Nat Rev Dis Primers. 2022; 8: 61. 36109590 10.1038/s41572-022-00389-z

[3] Kumar M Duraisamy K Chow BK Unlocking the non-IgE-mediated pseudo-allergic reaction puzzle with Mas-Related G-Protein Coupled Receptor Member X2 (MRGPRX2). Cells. 2021; 10: 1033. 33925682 10.3390/cells10051033 PMC8146469

[4] Che D Zhang T Zhang T Zheng Y Hou Y Geng S He L Clarithromycin-treated chronic spontaneous urticaria with the negative regulation of FcεRΙ and MRGPRX2 activation via CD300f. Int Immunopharmacol. 2022; 110: 109063. 35853276 10.1016/j.intimp.2022.109063

[5] Zuberbier T Abdul Latiff AH Abuzakouk M Aquilina S Asero R Baker D Ballmer-Weber B Bangert C Ben-Shoshan M Bernstein JA Bindslev-Jensen C Brockow K Brzoza Z Chong Neto HJ Church MK Criado PR Danilycheva IV Dressler C Ensina LF Fonacier L The international EAACI/GA LEN/EuroGuiDerm/APAAACI guideline for the definition, classification, diagnosis, and management of urticaria. Allergy. 2022; 77: 734–766. 34536239 10.1111/all.15090

[6] Bernstein JS Bernstein JA Lang DM Chronic spontaneous urticaria: current and emerging biologic agents. Immunol Allergy Clin North Am. 2024; 44: 595–613. 39389712 10.1016/j.iac.2024.07.001

[7] Zuberbier T Ensina LF Giménez-Arnau A Grattan C Kocatürk E Kulthanan K Kolkhir P Maurer M Chronic urticaria: unmet needs, emerging drugs, and new perspectives on personalised treatment. Lancet. 2024; 404: 393–404. 39004090 10.1016/S0140-6736(24)00852-3

[8] Roy S Chompunud Na Ayudhya C Thapaliya M Deepak V Ali H Multifaceted MRGPRX2: New insight into the role of mast cells in health and disease. J Allergy Clin Immunol. 2021; 148: 293–308. 33957166 10.1016/j.jaci.2021.03.049 PMC8355064

[9] Toscano A Elst J Van Gasse AL Beyens M van der Poorten ML Bridts CH Mertens C Van Houdt M Hagendorens MM Van Remoortel S Timmermans JP Ebo DG Sabato V Mas-related G protein-coupled receptor MRGPRX2 in human basophils: Expression and functional studies. Front Immunol. 2023; 13: 1026304. 36726977 10.3389/fimmu.2022.1026304 PMC9885256

[10] Wollam J Solomon M Villescaz C Lanier M Evans S Bacon C Freeman D Vasquez A Vest A Napora J Charlot B Cavarlez C Kim A Dvorak L Selfridge B Huang L Nevarez A Dedman H Brooks J Frischbutter S Inhibition of mast cell degranulation by novel small molecule MRGPRX2 antagonists. J Allergy Clin Immunol. 2024; 154: 1033–1043. 38971540 10.1016/j.jaci.2024.07.002

[11] Kolkhir P Ali H Babina M Ebo D Sabato V Elst J Frischbutter S Pyatilova P Maurer M MRGPRX2 in drug allergy: What we know and what we do not know. J Allergy Clin Immunol. 2023; 151: 410–412. 36089079 10.1016/j.jaci.2022.09.004 PMC9905269

[12] Vander Does A Ju T Mohsin N Chopra D Yosipovitch G How to get rid of itching. Pharmacol Ther. 2023; 243: 108355. 36739914 10.1016/j.pharmthera.2023.108355

[13] Elieh-Ali-Komi D Metz M Kolkhir P Kocatürk E Scheffel J Frischbutter S Terhorst-Molawi D Fox L Maurer M Chronic urticaria and the pathogenic role of mast cells. Allergol Int. 2023; 72: 359–368. 37210251 10.1016/j.alit.2023.05.003

[14] Trier AM Kim BS Structural insights into MRGPRX2: A new vision of itch and allergy. J Allergy Clin Immunol. 2022; 149: 1221–1222. 35090947 10.1016/j.jaci.2022.01.017

[15] Kumar M Choi YG Wong T Li PH Chow BKC Beyond the classic players: Mas-related G protein-coupled receptor member X2 role in pruritus and skin diseases. J Eur Acad Dermatol Venereol. 2025; 39: 476–486. 39044547 10.1111/jdv.20249 PMC11851267

[16] Keller JJ Cutaneous neuropeptides: the missing link between psychological stress and chronic inflammatory skin disease? Arch Dermatol Res. 2023; 315: 1875–1881. 36700961 10.1007/s00403-023-02542-4

[17] Tomaszewska K Słodka A Tarkowski B Zalewska-Janowska A Neuro-Immuno-Psychological aspects of chronic urticaria. J Clin Med. 2023; 12: 3134. 37176575 10.3390/jcm12093134 PMC10179371

[18] Nishimori N Toyoshima S Sasaki-Sakamoto T Hayama K Terui T Okayama Y Serum level of hemokinin-1 is significantly lower in patients with chronic spontaneous urticaria than in healthy subjects. Allergol Int. 2021; 70: 480–488. 34090787 10.1016/j.alit.2021.05.002

[19] Konstantinou GN Riedl MA Valent P Podder I Maurer M Urticaria and Angioedema: Understanding Complex Pathomechanisms to Facilitate Patient Communication, Disease Management, and Future Treatment. J Allergy Clin Immunol Pract. 2023; 11: 94–106. 36610760 10.1016/j.jaip.2022.11.006

[20] Giménez-Arnau AM DeMontojoye L Asero R Cugno M Kulthanan K Yanase Y Hide M Kaplan AP The pathogenesis of chronic spontaneous urticaria: the role of infiltrating cells. J Allergy Clin Immunol Pract. 2021; 9: 2195–2208. 33823316 10.1016/j.jaip.2021.03.033

[21] Kaplan A Lebwohl M Giménez-Arnau AM Hide M Armstrong AW Maurer M Chronic spontaneous urticaria: Focus on pathophysiology to unlock treatment advances. Allergy. 2023; 78: 389–401. 36448493 10.1111/all.15603

[22] Baldo BA Toxicities of opioid analgesics: respiratory depression, histamine release, hemodynamic changes, hypersensitivity, serotonin toxicity. Arch Toxicol. 2021; 95: 2627–2642. 33974096 10.1007/s00204-021-03068-2

[23] Chompunud Na Ayudhya C Ali H Mas-Related G Mas-Related G Protein-Coupled Receptor-X2 and Its Role in Non-immunoglobulin E-Mediated Drug Hypersensitivity. Immunol Allergy Clin North Am. 2022; 42: 269–284. 35469618 10.1016/j.iac.2021.12.003 PMC9674431

[24] Yang F Limjunyawong N Peng Q Schroeder JT Saini S MacGlashan D Dong X Gao L Biological screening of a unique drug library targeting MRGPRX2. Front Immunol. 2022; 13: 997389. 36341461 10.3389/fimmu.2022.997389 PMC9635925

[25] Kühn H Kolkhir P Babina M Düll M Frischbutter S Fok JS Jiao Q Metz M Scheffel J Wolf K Kremer AE Maurer M Mas-related G protein-coupled receptor X2 and its activators in dermatologic allergies. J Allergy Clin Immunol. 2021; 147: 456–469. 33071069 10.1016/j.jaci.2020.08.027

[26] Wang J Zhang Y Che D Zeng Y Wu Y Qin Q Wang N Baicalin induces Mrgprb2-dependent pseudo-allergy in mice. Immunol Lett. 2020; 226: 55–61. 32707128 10.1016/j.imlet.2020.07.006

[27] Choi BY Ye YM Role of Platelet-Activating Factor in the Pathogenesis of Chronic Spontaneous Urticaria. Int J Mol Sci. 2024; 25: 12143. 39596211 10.3390/ijms252212143 PMC11594505

[28] Andrades E Clarós M Torres JV Nonell L González M Curto-Barredo L Rozas-Muñoz E Gimeno R Barranco C Pujol RM Izquierdo I Giménez-Arnau AM New transcriptome and clinical findings of platelet-activating factor in chronic spontaneous urticaria: Pathogenic and treatment relevance. Biofactors. 2022; 48: 1284–1294. 35927787 10.1002/biof.1880

[29] Choi BY Yang EM Jung HW Shin MK Jo J Cha HY Park HS Kang HC Ye YM Anti-heat shock protein 10 IgG in chronic spontaneous urticaria: Relation with miRNA-101-5p and platelet-activating factor. Allergy. 2023; 78: 3166–3177. 37415527 10.1111/all.15810

[30] Yang F Guo L Li Y Wang G Wang J Zhang C Fang GX Chen X Liu L Yan X Liu Q Qu C Xu Y Xiao P Zhu Z Li Z Zhou J Yu X Gao N Sun JP Structure, function and pharmacology of human itch receptor complexes. Nature. 2021; 600: 164–169. 34789875 10.1038/s41586-021-04077-y

[31] Huang EJ Reichardt LF Trk receptors: roles in neuronal signal transduction. Annu Rev Biochem. 2003; 72: 609–642. 12676795 10.1146/annurev.biochem.72.121801.161629

[32] Chaki S Alkanfari I Roy S Amponnawarat A Hui Y Oskeritzian CA Ali H Inhibition of Orai channel function regulates Mas-Related G Protein-Coupled Receptor-Mediated responses in mast cells. Front Immunol. 2022; 12: 803335. 35126366 10.3389/fimmu.2021.803335 PMC8810828

[33] Lerner L Babina M Zuberbier T Stevanovic K Beyond Allergies-Updates on The Role of Mas-Related G-Protein-Coupled Receptor X2 in Chronic Urticaria and Atopic Dermatitis. Cells. 2024; 13: 220. 38334612 10.3390/cells13030220 PMC10854933

[34] Guo Y Ollé L Proaño-Pérez E Aparicio C Guerrero M Muñoz-Cano R Martín M MRGPRX2 signaling involves the Lysyl-tRNA synthetase and MITF pathway. Front Immunol. 2023; 14: 1154108. 37234172 10.3389/fimmu.2023.1154108 PMC10206166

[35] Bal G Schneikert J Li Z Franke K Tripathi SR Zuberbier T Babina M CREB Is Indispensable to KIT Function in Human Skin Mast Cells-A Positive Feedback Loop between CREB and KIT Orchestrates Skin Mast Cell Fate. Cells. 2023; 13: 42. 38201246 10.3390/cells13010042 PMC10778115

[36] Suzuki Y Liu S Ogasawara T Sawasaki T Takasaki Y Yorozuya T Mogi M A novel MRGPRX2-targeting antagonistic DNA aptamer inhibits histamine release and prevents mast cell-mediated anaphylaxis. Eur J Pharmacol. 2020; 878: 173104. 32320700 10.1016/j.ejphar.2020.173104

[37] Yang BG Kim AR Lee D An SB Shim YA Jang MH Degranulation of mast cells as a target for drug development. Cells. 2023; 12: 1506. 37296626 10.3390/cells12111506 PMC10253146

[38] Zhang Y Huang Y Dang B Hu S Zhao C Wang Y Yuan Y Liu R Fisetin alleviates chronic urticaria by inhibiting mast cell activation via MRGPRX2. J Pharm Pharmacol. 2023; 75: 1310–1321. 37410860 10.1093/jpp/rgad056

[39] Ding Y Dang B Wang Y Zhao C An H Artemisinic acid attenuated symptoms of substance P-induced chronic urticaria in a mice model and mast cell degranulation via Lyn/PLC-p38 signal pathway. Int Immunopharmacol. 2022; 113: 109437. 36403523 10.1016/j.intimp.2022.109437

[40] Chhiba KD Saini SS Emerging IgE and non-IgE targeted therapies for chronic urticaria. Ann Allergy Asthma Immunol. 2026; 136: P249–P256. 10.1016/j.anai.2025.11.008 PMC12874352 41270830

[41] Evommune, Inc. Evommune Presents Full Phase 2 Data for Oral MRGPRX2 Inhibitor EVO756 in Chronic Inducible Urticaria During Late-Breaker at EADV 2025 Congress. (2025-09-19)[2026-01-06]. https://www.prnewswire.com/news-releases/evommune-presents-full-phase-2-data-fororal-mrgprx2-inhibitor-evo756-in-chronic-inducible-urticaria-during-late-breaker-at-eadv-2025-congress-302560722.html.

